# Effect of perioperative repetitive transcranial magnetic stimulation on postoperative cognitive function and peripheral inflammation in elderly total knee arthroplasty patients: study protocol for a randomized controlled trial

**DOI:** 10.1186/s13063-025-09158-1

**Published:** 2025-10-15

**Authors:** Zhenhua Wu, Huan Tian, Sicheng Li, Hongfei Ren, Zizhe Yao, Cai Jiang, Xiaohua Ke, Dunbing Huang, Zhonghua Lin

**Affiliations:** 1 Encephalopathy and Rehabilitation Center, The Second Affiliated Hospital of Zhejiang Chinese Medical University, Hangzhou, 310005 Zhejiang China; 2 Department of Rehabilitation Medicine, School of Medicine, Shanghai Fourth People’s Hospital, Tongji University, Shanghai, 200434 China; 3 Shengli Clinical Medical College of Fujian Medical University, Fuzhou, 350001 China; 4 Rehabilitation Medicine Center, Fujian Provincial Hospital, Fuzhou, 350001 China

**Keywords:** Perioperative period, Repetitive transcranial magnetic stimulation, Postoperative cognitive dysfunction, Elderly, Total knee arthroplasty

## Abstract

**Background:**

Postoperative cognitive dysfunction (POCD) is a serious and common complication after total knee arthroplasty (TKA) in the elderly. Studies have suggested that repetitive transcranial magnetic stimulation (rTMS) can reduce the levels of tumor necrosis factor-alpha (TNF-α), interleukin-1 beta (IL-1β), and interleukin-6 (IL-6) inflammatory factors in the hippocampus, inhibit neuroinflammatory responses in the brain, and reduce the damage to synapses, thereby improving cognitive dysfunction. However, the effectiveness of rTMS for POCD remains to be explored. Therefore, the aim of this study is to treat POCD after TKA in the elderly with rTMS to evaluate the clinical efficacy of rTMS for POCD.

**Methods:**

This single-center, randomized, sham-controlled, assessor-blinded, parallel-group trial will enroll 207 elderly patients undergoing TKA and allocate them 1:1:1 to control group, active rTMS group, or sham rTMS group. All participants will receive standardized perioperative management. In addition, patients randomized to the active rTMS group will undergo stimulation of the left dorsolateral prefrontal cortex (DLPFC) at 10 Hz, 2000 pulses per session (5 s trains with 25 s inter-train intervals; 100% resting motor threshold), once daily for five consecutive pre-operative days. The sham rTMS group will follow the identical target and schedule, but the coil will be oriented perpendicular to the skull to avoid effective cortical stimulation. Primary outcome includes incidence of POCD at postoperative day seven (MoCA-based item-level definition). Secondary outcomes include changes in the MoCA total score and a prespecified cognitive subtest battery comprising the Digit Span Test (DST), Digit Symbol Substitution Test (DSST), Trail Making Test (TMT), and Delayed Story Recall (DSR), together with changes in serum inflammatory markers (IL-1β, IL-6, TNF-α, and HMGB1), assessed at preoperative day six, preoperative day one, postoperative day three, and postoperative day seven.

**Discussion:**

This trial will contribute to addressing the effectiveness of perioperative rTMS stimulation in elderly patients with TKA, and will initially clarify that perioperative rTMS preventive intervention can produce neuroprotective effects to reduce oxidative stress and inflammation, and to some extent block the possible pathway of POCD occurrence, thereby reducing the risk of POCD occurrence.

**Trial registration:**

ClinicalTrials.gov ChiCTR2400081372. Registered on 29 February 2024.

**Supplementary Information:**

The online version contains supplementary material available at 10.1186/s13063-025-09158-1.

## Administrative information

Note: the numbers in curly brackets in this protocol refer to SPIRIT checklist item numbers. The order of the items has been modified to group similar items (see http://www.equator-network.org/reporting-guidelines/spirit-2013-statement-defining-standard-protocol-items-for-clinical-trials/).

**Table:** 

Title {1}	Effect of perioperative repetitive transcranial magnetic stimulation on postoperative cognitive function and peripheral inflammation in elderly total knee arthroplasty patients: study protocol for a randomized controlled trial
Trial registration {2a and 2b}.	Chinese Clinical Trial Registry (ChiCTR2400081372); registered on 29 February 2024.
Protocol version {3}	Version 1.0, dated 1 April 2024
Funding {4}	This project is supported by the Key Discipline Construction Program of Traditional Chinese Medicine in China, the Hongkou District Health Commission Medical Research Project (Grant number: Hongwei 2302-14), the Central Leading Local Science and Technology Development Special Fund Project (Grant number: 2023L3018), the Guiding Projects of Fujian Science and Technology Department (Grant number:2023Y0101), and the Key Discipline Construction Program of Traditional Chinese Medicine in Fujian Province.
Author details {5a}	ZHW, DBH. Encephalopathy and Rehabilitation Center, The second Afiliated Hospital of Zhejiang Chinese Medical University, Hangzhou, 310005, China HT, SCL, HFR, ZZY, XHK. Department of Rehabilitation Medicine, School of Medicine, Shanghai fourth People’s Hospital, Tongji University, shanghai, 200434, China CJ, ZHL. Shengli Clinical Medical College of Fujian Medical University, Fuzhou, 350001, China; Rehabilitation Medicine Center, Fujian Provincial Hospital, Fuzhou, 350001, China
ame and contact information for the trial sponsor {5b}	ZHL Shengli Clinical Medical College of Fujian Medical University, Fuzhou, 350001, China. doctor Izh71@163.com XHK Department of Rehabilitation Medicine, Shanghai Fourth People's Hospital, School of Medicine, Tongji University, Shanghai, 200434, China. kxh22@tongji.edu.cn ZHW Encephalopathy and Rehabilitation Center, The second Afiliated Hospital of Zhejiang Chinese Medical University, Hangzhou, 310005, China. 458800977@qq.com
Role of sponsor {5c}	The ZHW, XHK and ZHL provided overall oversight for regulatory and administrative compliance. The funders listed under Funding {4} provided financial support only and had no role in the study design; collection, management, analysis, or interpretation of data; writing of the report; or the decision to submit the manuscript for publication. The investigators retained full access to the data and full responsibility for the content and the decision to publish.

## Introduction

### Background and rationale {6a}

Osteoarthritis of the knee is a highly disabling chronic joint disease with a prevalence of approximately 50% in people over 60 years of age and more than 80% in people over 75 years of age [1]. End-stage osteoarthritis of the knee is accompanied by severe joint degeneration, which has a significant impact on the patient’s quality of life, leading to chronic pain, functional limitations, and disability. Total knee arthroplasty (TKA) is an effective and economical treatment for end-stage knee osteoarthritis [2]. Although TKA is the most effective treatment, it is also considered one of the most invasive and painful orthopedic surgeries, which predisposes elderly patients to a variety of postoperative complications [3, 4]. Among them, postoperative cognitive dysfunction (POCD) is a common postoperative central nervous system complication, which is mainly manifested as a decrease in learning ability, memory function, and information processing ability and speed, in addition to personality changes and a decrease in social activities, accompanied by central nervous system damage [5]. Furthermore, POCD may lead to an increase in postoperative complications, prolong patients’ hospital stays, increase the burden on families and society, and seriously affect patients’ postoperative quality of life [6]. Up to now, the treatment of POCD is based on preoperative cognitive training, pharmacological intervention, intraoperative management, and pain management, and there is no particular treatment for POCD [7]. Therefore, elderly TKA patients are facing a serious risk of POCD, and it is urgent to find appropriate interventions to reduce the incidence of POCD in elderly TKA patients.

In a previous study, preoperative probiotic supplementation was found to be effective in reducing POCD in elderly patients after TKA [8]. It suggested that perioperative prophylactic intervention in our perioperative period can block the possible pathway of POCD occurrence to a certain extent. It is suggested that perioperative prophylactic intervention may block the pathway of POCD occurrence to some extent. However, the exact pathogenic mechanism of POCD is still unclear. In recent years, some studies have suggested that the inflammatory response has a central role in the development of POCD [9]. Moreover, it has been shown that levels of interleukin-1 beta (IL-1β), interleukin-6 (IL-6), and tumor necrosis factor-alpha (TNF-α) are elevated in both the peripheral and central nervous system after surgery in patients with POCD, and the degree of elevation is highly correlated with the severity and duration of POCD [10]. Additionally, surgery could promote the release and activation of high-mobility group box 1 (HMGB1). The activated HMGB1 participates in the innate immune response by binding to receptors on circulating bone marrow-derived monocytes and increases the synthesis and release of proinflammatory cytokines into the circulation, destroying the blood–brain barrier [11]. Importantly, the level of HMGB1 in peripheral blood is strongly associated with the development of POCD [12]. On this mechanistic background, targeting prefrontal control networks to modulate inflammatory signaling represents a plausible preventive strategy.

Repetitive transcranial magnetic stimulation (rTMS) is a painless and non-invasive neuromodulation technique. It can not only regulate nerve excitability and cortical function, but also modulate the activity of individual neurons [13], and it has been widely used in the treatment of a wide range of neuropsychiatric disorders. Studies have shown that cytokines (such as IL-1β, IL-6, and TNF-α) play a significant role in the pathophysiology of cognitive dysfunction. Moreover, rTMS has shown potential in regulating cognitive function by targeting the neuroinflammatory pathways associated with postoperative cognitive dysfunction (POCD). Recent studies exploring the effects of rTMS on inflammatory markers (such as IL-6 and TNF-α) also support its potential in alleviating neuroinflammation [14–16]. We hypothesize that, compared with sham rTMS and standardized perioperative management, perioperative left DLPFC rTMS will improve early postoperative cognition and attenuate perioperative increases in inflammatory biomarkers (IL-1β, IL-6, TNF-α, HMGB1). Accordingly, we designed a randomized, sham-controlled trial to test whether perioperative rTMS can improve cognition and modulate inflammatory responses after TKA.

### Objectives {7}

1. To investigate the effect of perioperative rTMS on postoperative cognitive function in elderly patients undergoing TKA.
2. To test the hypothesis that POCD is closely related to inflammatory responses (especially activation of inflammatory factors IL-1β, IL-6, TNF-a, and HMGB1), and to investigate the role of perioperative rTMS prophylactic interventions in reducing oxidative stress and inflammation, and lowering the levels of inflammatory factors in delaying POCD.

### Trial design {8}

**Fig. 1:**
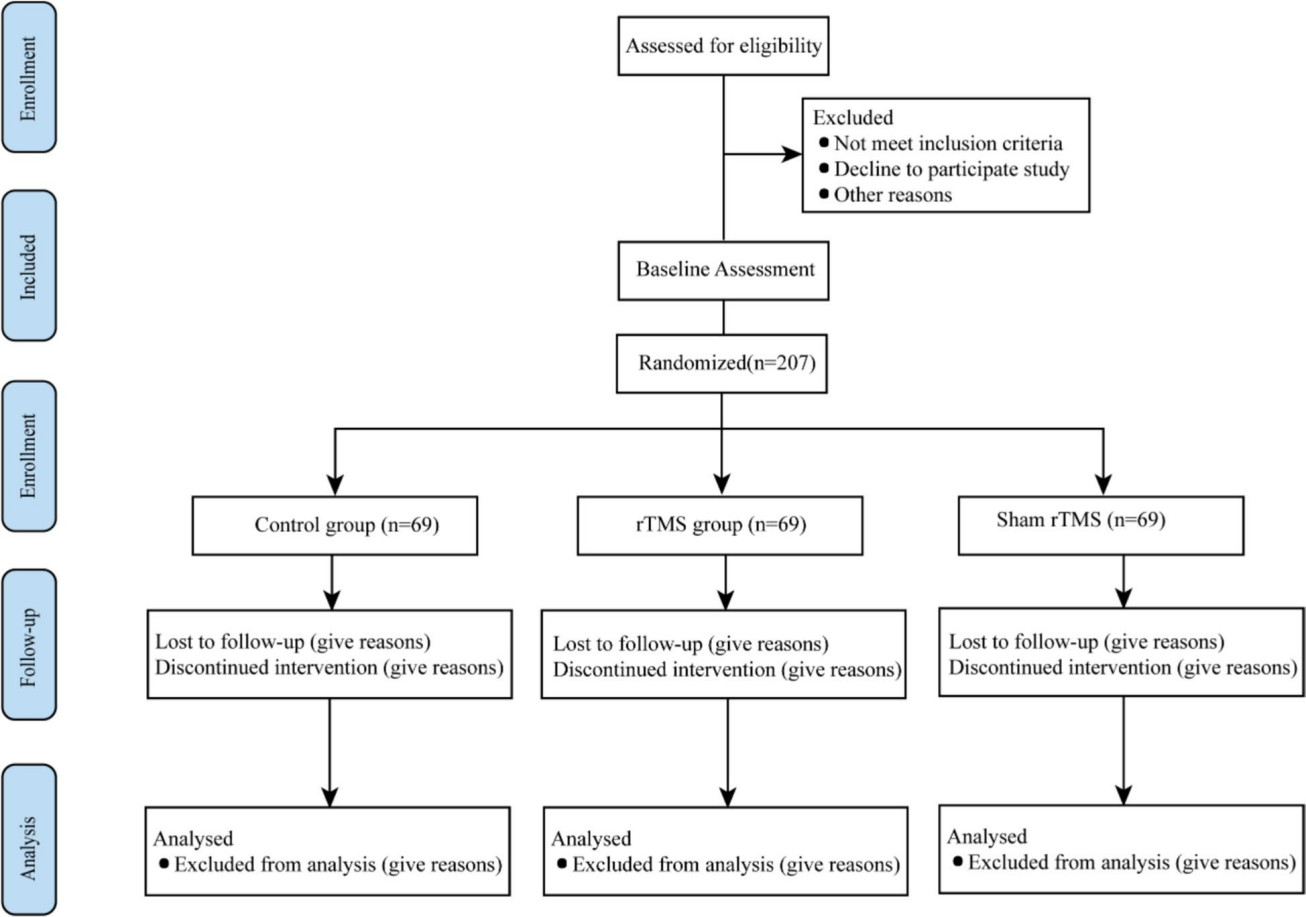
Flowchart of the research procedure

**Fig. 2:**
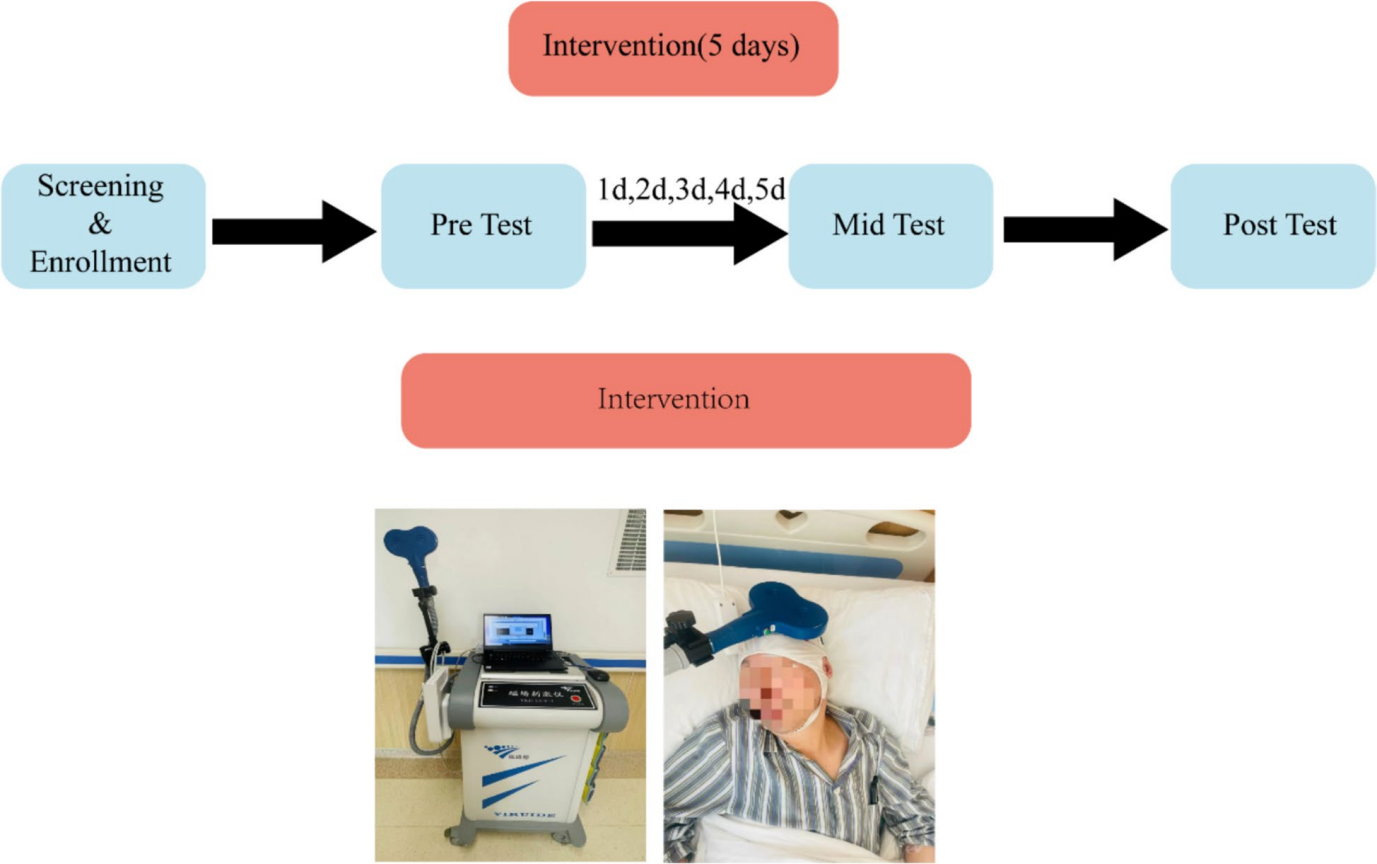
Flowchart of intervention and assessment

This single-center, randomized, sham-controlled, assessor-blinded, three-arm parallel-group trial will allocate participants to (1) standardized perioperative management (control), (2) active rTMS, or (3) sham rTMS. Active rTMS will be compared with sham rTMS and with standardized perioperative management during the perioperative period in elderly patients undergoing TKA. All participants will receive a standardized perioperative pathway comprising pre-operative functional assessment and quadriceps-strengthening exercises; intra-operative combined spinal–epidural anesthesia with tourniquet use and tranexamic acid; and post-operative multimodal analgesia with early mobilization. The overall study flow is shown in Fig. 1. The SPIRIT schedule of enrollment, interventions, and assessments (including timing of the five preoperative rTMS/sham sessions and all outcome timepoints) is presented in Table 1. The detailed intervention and assessment flowchart is provided in Fig. 2.

**Table 1:**
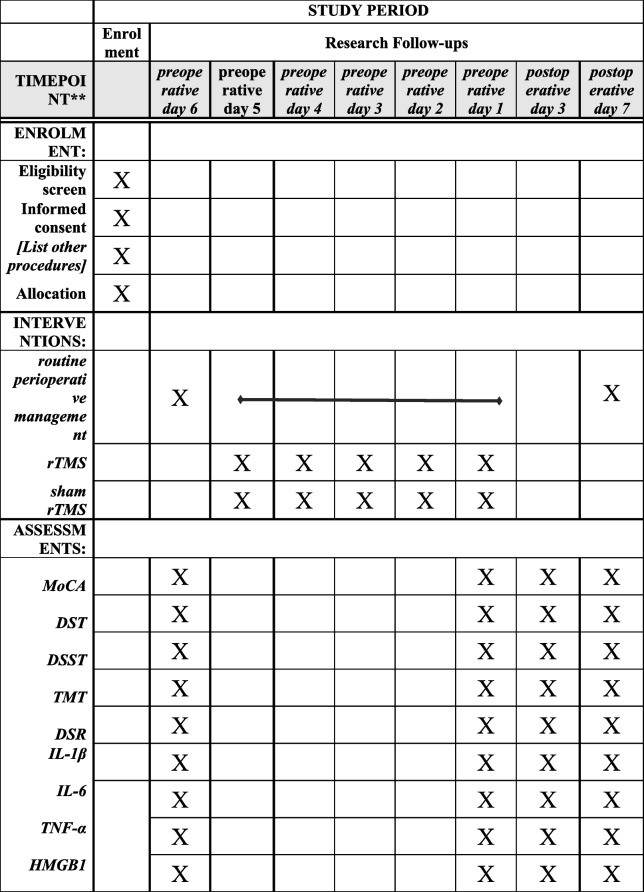
SPIRIT schedule of enrolment, interventions, and assessments

Standard Protocol Items: Recommendations for Interventional Trials (SPIRIT) figure of enrolment, interventions and assessments

*rTMS* Repetitive transcranial magnetic stimulation, *MoCA* Montreal Cognitive Assessment, *DST* Digit span test, *DSST* Digit symbol substitution test, *TMT* Trail making test, *DSR* Delayed story recall, *CAM-CR* Confusion assessment method-Chinese reversion, *IL-1β* Interleukin-1beta, *IL-6* Interleukin 6, *TNF-a* Tumor necrosis factor-alpha; HMGB1, high-mobility group box 1

## Methods: participants, interventions, and outcomes

### Study setting {9}

Patients who are scheduled to undergo TKA patients will be recruited to the Shanghai Fourth People’s Hospital. Patients who meet the following criteria will be considered for inclusion in the study.

### Eligibility criteria {10}

#### Inclusion criteria

The inclusion criteria are as follows: (1) age ≥ 60 years, male or female; (2) needed to be performed TKA due to knee osteoarthritis; (3) American Society of Anesthesiologists grade I to II; (4) Preoperative Montreal Cognitive Assessment (MoCA) ≥ 26 points; (5) normal speech and hearing, able to co-operate with rehabilitation assessment and rehabilitation therapy.

#### Exclusion criteria

The exclusion criteria will be following: (1) drug allergy, severe cardiovascular disease, hepatic or renal dysfunction; (2) use of sedative-hypnotics or medications with anticholinergic effects that cannot be discontinued at least 4 weeks prior to baseline; (3) contraindications to rTMS (e.g., history of epilepsy, cochlear implant recipients, patients with metal implants in the head or pacemakers in the heart); (4) those who are participating in other clinical trials; (5) postoperative complications such as lung 34 infection, pulmonary embolism, or stroke; (6) personal or legal representatives unable or unwilling to give written informed consent.

#### Withdrawal criteria 

The Withdrawal criteria will be the following: (1) those who failed to follow the trial protocol treatment; (2) those who do not wish to continue in the trial; (3) those whose condition has worsened after the start of the trial or who have had a serious adverse event that makes continuation of the trial inadvisable.

### Who will take informed consent? {26a}

Based on the above criteria, to confirm that a patient undergoing TKA has been screened for eligibility to participate in this study, the treatment team has assessed the patient as eligible, and the surgeon will complete the initial study information for the patient. The patient is invited to meet with the investigator and physician to discuss any remaining questions and sign an informed consent form.

The Informed Consent Form is provided as Supplementary File S1. These templates cover study purpose/procedures, risks and benefits (including rTMS-related risks), alternatives, confidentiality and data handling, biospecimen use and destruction, compensation/contacts, and the voluntary right to withdraw without affecting care.

### Additional consent provisions for collection and use of participant data and biological specimens {26b}

In this study, participants’ biospecimens will be collected and patients will be informed of the purpose of biospecimen collection and possible privacy risks, and consent will be obtained from the participants.

## Interventions

### Explanation for the choice of comparators {6b}

The control group received standardized perioperative management.

### Intervention description {11a}

#### Control group

The control group will be subjected to standardized perioperative management. Specific measures are as follows: (1) Preoperative phase: patients will undergo a standardized perioperative pathway comprising functional assessment to inform care and prehabilitation focusing on quadriceps-strengthening exercises; pharmacological venous thromboembolism (VTE) prophylaxis will be administered as per institutional protocol. All eligibility screening is detailed under the “Eligibility criteria {10}” and “Recruitment {15}” sections. (2) Intraoperative phase: the procedure was performed under spinal-epidural anesthesia. A medial parapatellar approach was utilized for component implantation. A tourniquet, inflated to 250–300 mm Hg, and intravenous tranexamic acid (15 mg/kg) were employed to reduce perioperative blood loss. (3) Postoperative phase: pain management involved a multimodal regimen incorporating femoral nerve blocks and oral analgesics (acetaminophen/celecoxib). VTE prophylaxis was continued with intermittent pneumatic compression and rivaroxaban (10 mg/day). Early mobilization was enforced, with ambulation commencing on the first postoperative day. Patients were monitored daily for complications, including surgical site infection, VTE, and neurovascular injury.

#### Active rTMS group

The active rTMS group will be given active rTMS treatment based on standardized perioperative management. Active rTMS will be delivered using a magnetic stimulator (YRD-CCY-1, YIRUIDE Medical Equipment, Wuhan, China) with a figure-of-eight 70-mm coil positioned tangential to the scalp over the left dorsolateral prefrontal cortex (DLPFC; 10–20 system F3). Stimulation will be applied at 10 Hz in 5-s trains with 25-s inter-train intervals, 2000 pulses per session, at an intensity of 100% of the resting motor threshold (RMT). RMT will be defined as the lowest stimulator output that elicits motor-evoked potentials (MEPs) ≥ 50 μV in the relaxed right abductor pollicis brevis in ≥ 5 of 10 trials. Sessions will be delivered once daily for five consecutive pre-operative days. No post-operative rTMS will be delivered in this protocol; surgery is scheduled within 24–48 h after the final pre-operative session. Participants will be continuously monitored; if intolerable local discomfort occurs, stimulation will be stopped immediately and documented.

Rationale for stimulation site: The left dorsolateral prefrontal cortex (DLPFC) is a hub of executive-control and working-memory networks implicated in early postoperative cognition. Against the peri-operative neuroinflammatory background relevant to POCD (e.g., IL-1β, IL-6, TNF-α, HMGB1), prefrontal neuromodulation offers a biologically plausible route to support cognition and modulate inflammatory signaling. Accordingly, the left DLPFC (F3) was selected as the stimulation target.

#### Sham rTMS group

The sham rTMS group will be given sham rTMS treatment based on standardized perioperative management. The stimulation site, stimulation parameters, and treatment schedule of the sham rTMS treatment will all be the same as the active rTMS group, and the patient will be able to hear the sound of the magnetic stimulator. However, the coil will be placed perpendicular to the skull surface.

### Criteria for discontinuing or modifying allocated interventions {11b}

Participant-level criteria and actions: An rTMS/sham session will be interrupted or discontinued if any of the following occur during or immediately after stimulation: (1) intolerable scalp discomfort or pain despite repositioning; (2) vasovagal syncope or pre-syncope; (3) sustained hypertension (> 180/110 mmHg), tachycardia (> 130 bpm) or clinically significant arrhythmia; (4) new focal neurological symptoms/signs; (5) any seizure; (6) coil-related skin heating/burn; or (7) persistent severe headache not relieved by simple analgesia, or any other event judged clinically significant and related to the procedure by the investigator. The session will be stopped, clinical care provided, and the event documented and reported per the Harms procedures (see {22}). Resumption of stimulation at a reduced intensity and/or fewer trains may be considered after resolution of symptoms at the investigator’s discretion. Occurrence of a seizure or any unanticipated serious adverse device effect will lead to permanent discontinuation of rTMS for that participant. Participants may also withdraw from the intervention at any time; unless consent is withdrawn, they will remain in outcome follow-up (intention-to-treat).

Trial-level pausing or termination: Decisions to pause, modify, or terminate the trial will be guided by the independent DSMB according to pre-specified safety triggers and overall safety review (see Interim analyses and stopping guidelines {21b} and Harms {22}). Illustrative triggers include: any definite device-related seizure; ≥ 2 rTMS-related SAEs or an rTMS-related SAE rate > 5% and exceeding the sham arm by ≥ 5 percentage points; any unanticipated serious adverse device effect; or a clear excess of all-cause SAEs in the active rTMS arm at a scheduled review. The DSMB will issue recommendations to the TSC and Sponsor/PI; final operational decisions will be made by the Sponsor/PI upon TSC advice.

### Strategies to improve adherence to interventions {11c}

The rTMS treatment program is known for its high degree of flexibility and safety and can respond to and adapt to the individual needs of the patient, thus ensuring a very high program compliance rate. The entire rTMS treatment process will be carried out under the strict supervision of our hospital’s experienced and professional rehabilitation therapists to ensure the professionalism, safety, and effectiveness of the treatment process.

### Relevant concomitant care permitted or prohibited during the trial {11d}

Perioperative use of medications that substantially affect cognition (e.g., benzodiazepine sleep aids, anticholinergic antiemetics/antihistamines, and high-dose antipsychotics for non-delirium indications) will be prohibited unless clinically indispensable and the expected benefits outweigh the risks to study validity. The anesthesia and surgical teams will follow a standardized, enhanced recovery after surgery (ERAS)-consistent pathway to minimize such exposure; where antiemetic or sedative and analgesic therapy is required, non-anticholinergic alternatives (e.g., ondansetron) and opioid-sparing strategies will be preferred; meperidine (pethidine) will be avoided. Any rescue use of prohibited/limited agents will be prospectively recorded (drug, dose, frequency, duration, timing, indication) as a protocol deviation without excluding the participant from intention-to-treat analyses. Medication exposure will be captured during hospitalization and at discharge. Quantitative exposure metrics—including total opioid burden expressed as morphine milligram equivalents (MME) and a composite psychotropic medication score—will be incorporated as covariates in the primary regression models to adjust for potential confounding. A prespecified sensitivity analysis will exclude participants with extreme exposure to potentially confounding medications to assess robustness.

### Provisions for post-trial care {30}

Waiting for the end of the experiment, the control group will receive 5 rTMS treatments free of charge. If other symptoms of the subjects worsen during the study, rTMS treatment should be stopped immediately and symptomatic treatment should be provided free of charge, as well as psychological counseling for the patients. If the injury is serious, the relevant specialists will be invited to treat the injury and compensate for the medical expenses.

### Outcomes {12}

#### Primary outcome (five-element specification)

Domain: POCD.

Specific measurement: MoCA administered at four scheduled assessments (preoperative day six, preoperative day one (baseline), postoperative day three, postoperative day seven) by a trained, blinded assessor.

Metric: Binary classification of POCD at each postoperative assessment. POCD is defined a priori as a ≥ 20% decline from baseline in ≥ 2 MoCA items (Newman criteria) [17].

Method of aggregation: The group-level proportion with POCD at postoperative day seven (primary time point); between-group effect expressed as risk ratio with 95% CI from a log-binomial model (logistic regression if non-convergent), adjusted for age, sex, education, and baseline MoCA.

Time point: postoperative day seven (primary); postoperative day three analyzed as supportive.

#### Secondary outcomes (five-element specification)

MoCA total score—measurement: total score (0–30); metric: change from baseline; aggregation: mean (SD) and mean difference (95% CI) from linear mixed models; time points: preoperative day six, preoperative day one, postoperative day three, postoperative day seven.

Digit Span Test (DST) (forward/backward)—measurement per standard DST; metric: value and change; aggregation: mean (SD) and mean difference; time points: preoperative day six, preoperative day one, postoperative day three, postoperative day seven.

Digit Symbol Substitution Test (DSST)—measurement: number correct in 90 s; metric: value and change; aggregation: mean (SD) and mean difference; time points: preoperative day six, preoperative day one, postoperative day three, postoperative day seven.

Trail Making Test (TMT)—measurement: time to completion (s); metric: value and change; aggregation: mean (SD); if skewed, median (IQR) and Hodges–Lehmann difference (pre-specified); time points: preoperative day six, preoperative day one, postoperative day three, postoperative day seven.

Delayed Story Recall (DSR)—measurement: story recall points; metric: value and change; aggregation: mean (SD) and mean difference; time points: preoperative day six, preoperative day one, postoperative day three, postoperative day seven.

Inflammatory markers (IL-1β, IL-6, TNF-α, HMGB1)—measurement: serum ELISA (pg/mL); metric: log-change from baseline; aggregation: geometric mean ratio (ANCOVA, baseline-adjusted) (or mean difference if normal); time points: preoperative day six, preoperative day one, postoperative day three, postoperative day seven.

All time points conform to the pre-specified SPIRIT schedule (preoperative day six, preoperative day one, postoperative day three, postoperative day seven); statistical baseline is defined as pre-op day 1 for all change analyses.

### Participant timeline {13}

Following enrollment, each outcome indicator will be assessed at four time points: before the rTMS intervention (on preoperative day six), after the completion of the rTMS intervention (on preoperative day one), on postoperative day three, and on postoperative day seven. The schedule of enrollment, interventions, and assessments is summarized in the Standard Protocol Items: Recommendations for Interventional Trials (SPIRIT) figure.

### Sample size {14}

Based on previous studies, we assumed a 40% incidence of POCD in elderly patients undergoing knee replacement [5], and following Rodriguez et al. (2005), considered a clinically meaningful absolute reduction of 15 percentage points [18]. With three groups (control, active rTMS, sham) allocated 1:1:1, 80% power, and a two-sided *α* = 0.05, 186 participants are required to detect this difference. Allowing for an anticipated 10% attrition, the planned sample size is 207. This inflation accounts for expected loss to follow-up; if attrition exceeds this allowance and threatens statistical power, the TSC may, on DSMB recommendation and blinded operational data, approve extending recruitment to preserve power without unblinding efficacy.

### Recruitment {15}

Recruitment will take place at Shanghai Fourth People’s Hospital during the elective TKA pathway. Potential participants will be pre-screened at the pre-assessment clinic and on the orthopedic ward against the eligibility criteria (e.g., radiographic severity consistent with Kellgren–Lawrence grade ≥ 3), with written informed consent obtained prior to allocation. Recruitment strategies include surgeon referral at pre-assessment clinics, ward-based screening, and patient-facing materials (posters and flyers). Accrual numbers will be reported only under Trial status; detailed inclusion/exclusion criteria are provided in the “Eligibility criteria {10}” section.

### Patient and public involvement

A Patient Advisory Panel (PAP), comprising two older adults who previously underwent total knee arthroplasty and one caregiver, was consulted during the design phase. Their feedback improved the clarity and accessibility of the participant information sheet and consent form and helped optimize the feasibility and burden of the assessment schedule. During the trial, the PAP will provide consultative input quarterly (start of accrual, mid-study, and prior to database lock), focusing on participant burden, acceptability of procedures, and the preparation of a lay summary of results for dissemination. The PAP has an advisory role only: members will not access identifiable data, will not participate in outcome assessment, statistical analyses, or operational decision-making, and will follow confidentiality requirements. To support meaningful involvement, the study team will provide a brief orientation and plain-language summaries of study procedures and interim progress. Reasonable expenses for attendance will be reimbursed according to institutional policy. PAP contributions will be acknowledged in study outputs; no authorship is conferred by advisory activities.

## Assignment of interventions: allocation

### Sequence generation {16a}

The randomization sequence will be conducted by SAS statistical software version 9.4 (SAS Institute Inc. Cary, NC) by an independent researcher.

### Concealment mechanism {16b}

The researcher responsible for recruitment will assign the eligibility number and then receive a closed, opaque envelope containing the randomization number, allocation, and intervention information.

### Implementation {16c}

The subject assignment will be concealed by placing the assigned tasks in sequentially numbered, opaque sealed envelopes. Clinicians will determine whether subjects are available to participate in the study in strict accordance with the above inclusion and exclusion criteria. The researcher conducting the clinical intervention will open the envelope and be informed of the intervention plan to be delivered to the participant.

## Assignment of interventions: blinding

### Who will be blinded {17a}

Participants, outcome assessors, and statisticians will remain blinded to group allocation. rTMS operators are unblinded due to the nature of the procedure. Blinding success will be evaluated by end-of-treatment allocation-guess questionnaires.

### Procedure for unblinding if needed {17b}

Emergency unblinding is permitted only when knowledge of allocation is essential for clinical management; procedures are held by an independent data manager.

## Data collection and management

### Plans for assessment and collection of outcomes {18a}

All trial personnel will complete role-specific training prior to participant enrollment and will be certified by the PI or delegate. (1) rTMS operators: device operation; coil positioning (F3); motor-threshold determination; stimulation parameters; contraindication screening; and emergency procedures (e.g., syncope/seizure management). (2) Outcome assessors (blinded): standardized administration and scoring of MoCA, DST, DSST, TMT, and DSR with inter-rater checks; maintenance of blinding and separation from intervention delivery. (3) Biospecimen handling staff: venepuncture; processing (centrifugation, aliquoting); − 80 °C storage; ELISA SOPs; chain-of-custody and labeling. (4) AE/SAE monitoring: definitions, reporting timelines, documentation pathways, and escalation to the DSMB per {22}, including immediate reporting and predefined management plans for any adverse reactions.

Training will be documented in logs (date, content/SOP IDs, trainer/trainee signatures), with refresher sessions at least annually or after protocol amendments. Protocol adherence will be monitored through periodic audits and team meetings. During screening and before the first rTMS session, participants will complete a standard rTMS safety questionnaire and undergo physical and neurological examinations to confirm eligibility and safety (see {10}/{11a}).

### Plans to promote participant retention and complete follow-up {18b}

During recruitment, participants will receive comprehensive information on study procedures, requirements, and the importance of completing follow-up. Participants may discontinue rTMS at any time without obligation to provide a reason; withdrawal from the intervention does not constitute withdrawal from follow-up unless consent is revoked. To enhance retention and minimize missing data, we will use multi-modal reminders (phone, text, and written), provide reminders to complete questionnaires at each study visit, offer flexible assessment windows around each time point, and, where feasible, arrange transport assistance. All contact attempts and outcomes will be prospectively logged.

### Data management {19}

Patient data is centrally collected through electronic case report form (eCRF); data is securely protected, and study folders are backed up monthly. Informed consent forms were filed electronically, and paper copies were stored securely. Information on adverse events (AEs) and serious adverse events (SAEs) was entered into 17–34 eCRF for management. All data modifications and analysis steps were documented in detail in eCRF and SPSS, and source data were retained in the electronic medical record. At the end of the study, all data, including patient information, were archived for 30 years to ensure long-term accessibility and compliance.

### Confidentiality {27}

In this study, all participant data will be stored in an anonymized form, which is achieved by assigning a unique study identifier to each participant. The keys to these identifiers will be strictly confined within the research team for the duration of the study and will be properly documented and handled confidentially by the Principal Investigator upon completion of the study based on strict research protocols. It is ensured that no specific information that can identify the patient will be disclosed in the publicly published results.

It is important to note that plans for the collection, laboratory testing, and storage of biological specimens required for genetic and molecular analyses that may be involved in this trial or in the future are not applicable here. Biological samples collected for this study are limited to being used as a basis for safety assessment and are expressly excluded from being used for any form of genetic or molecular research analysis. Further, once the biospecimens have fulfilled their intended use in safety monitoring, they will be immediately and securely destroyed, a move that not only protects the privacy interests of the participants but also strictly adheres to the highest standards of medical ethics.

### Plans for collection, laboratory evaluation and storage of biological specimens for genetic or molecular analysis in this trial/future use {33}

Not applicable because there is no plan for collection, laboratory evaluation, and storage of biological specimens for genetic or molecular analyses that will be used in this trial/in the future.

### Statistical methods

#### Statistical methods for primary and secondary outcomes {20a}

Analyses will follow intention-to-treat. Primary endpoint (POCD at postoperative day seven) will be compared between groups using log-binomial regression to estimate risk ratios (95% CI), adjusted for age, sex, education, and baseline MoCA; logistic regression will be used if convergence issues arise. A supportive analysis will model POCD trajectories at postoperative day three and postoperative day seven using generalized estimating equations with a log link.

Continuous secondary outcomes (MoCA total, DST, DSST, TMT, DSR) will be analyzed using linear mixed-effects models (fixed effects for group, time, and group × time; random intercept for participant), adjusting for baseline. For TMT, if residuals are markedly non-normal, we will report median (IQR) and between-group Hodges–Lehmann differences as pre-specified sensitivity analyses. Inflammatory markers will be log-transformed and analyzed using ANCOVA at each post-op time point with baseline adjustment; results will be back-transformed as geometric mean ratios (95% CI). Missing data will be handled under a missing-at-random assumption via maximum likelihood; multiple imputation (*m* = 20) will be a sensitivity analysis. Multiplicity across secondary outcomes will be controlled by the Benjamini–Hochberg procedure (false discovery rate 5%).

### Interim analyses and stopping guidelines {21b}

No formal interim efficacy analyses are planned. The rationale is threefold: (1) Endpoint timing and stability: The primary endpoint is the short-term change in cognitive performance at postoperative day 7; early looks would be based on small, potentially unstable samples and risk spurious findings without materially altering perioperative care. (2) Statistical operating characteristics: This is a three-arm trial with a modest total sample size. Alpha spending to accommodate interim efficacy looks would reduce the final power or require complex multiplicity control, which is not justified given the endpoint and effect-size uncertainty. (3) Bias minimization and trial integrity. Avoiding interim efficacy looks reduces operational and analytical bias risks (e.g., recruitment drift, performance bias) and preserves blinding and data quality.

Safety oversight and stopping for harm: An independent DSMB will review unblinded safety data six-monthly and ad hoc if signals emerge. The DSMB may recommend a temporary pause or early termination for safety if any pre-specified trigger is met, including: (1) any definite device-related seizure; (2) ≥ 2 rTMS-related SAEs or an rTMS-related SAE rate > 5% and exceeding the sham arm by ≥ 5 percentage points; (3) any unanticipated serious adverse device effect (USADE); or (4) clear excess of all-cause SAEs in the active rTMS arm versus sham at a scheduled review (conservative threshold). Final decisions will be made by the Sponsor/PI upon TSC advice.

### Methods for additional analyses (e.g., subgroup analyses) {20b}

There are no subgroup analyses planned.

### Methods in analysis to handle protocol non-adherence and any statistical methods to handle missing data {20c}

Analyses will follow the intention-to-treat principle, retaining all randomized participants in their allocated groups. To minimize loss to follow-up, we will employ proactive retention strategies (appointment reminders, flexible assessment windows, and phone/text follow-ups). For the primary endpoint and other repeated continuous outcomes, linear mixed-effects models will be estimated under a missing-at-random assumption using maximum likelihood; results will be stress-tested by prespecified sensitivity analyses including multiple imputation (*m* = 20) and a conservative worst-case scenario. Participants who deviate from the intervention protocol will remain in the ITT analysis; per-protocol/sensitivity analyses may be reported descriptively. We will not replace randomized participants; if attrition exceeds the prespecified allowance and materially threatens power, the TSC may, on DSMB advice and blinded aggregate information, approve extending recruitment to preserve target precision.

### Plans to give access to the full protocol, participant level-data and statistical code {31c}

The datasets used and/or analyzed in this study may be provided by the corresponding author(s) upon reasonable request and subject to agreement with the Shanghai Fourth People’s Hospital’s guidelines for research collaboration and data transfer.

### Oversight and monitoring

#### Composition, roles, responsibilities, and meeting schedules of coordinating and oversight bodies {5d}

1. Trial Coordination Centre (TCC): The TCC, based at the Department of Rehabilitation Medicine, Shanghai Fourth People’s Hospital, provides day-to-day operational management and support. Composition: principal investigator, trial manager, research assistant/coordinator, and two data managers. Responsibilities: (i) overall coordination to ensure conduct per protocol, Good Clinical Practice (GCP), and regulatory requirements; (ii) site operations (screening/enrolment on the orthopaedic ward, scheduling active/sham rTMS, integration with routine surgical workflows); (iii) data management (collection, entry, quality control, security, dataset preparation for the independent statistician); (iv) safety monitoring (collection and initial documentation of all AEs/SAEs, notification to the PI and DSMB); and (v) communications (single point of contact for trial-related queries).

Meeting frequency: the full TCC meets weekly; ad-hoc huddles may be convened when operational issues arise between scheduled meetings.

(2) Trial Steering Committee (TSC): An independent TSC (independent clinical trialist, independent statistician, and geriatric medicine advisor) provides overall oversight of progress, protocol adherence, and considers DSMB recommendations.

Meeting frequency: every 6 months, or ad hoc if triggered by safety/operational concerns.

Reporting: the TCC submits blinded progress reports to the TSC; the TSC issues oversight recommendations to the Sponsor/PI.

(3) Data and Safety Monitoring Board (DSMB): An independent DSMB (neurologist/rTMS expert and anaesthesiologist) reviews unblinded safety data to safeguard participants and trial integrity. No formal interim efficacy analyses are planned.

Meeting frequency: every 6 months for scheduled reviews, and ad hoc if safety signals emerge.

(4) Patient and Public Involvement (PPI) pointer: A Patient Advisory Panel (two prior TKA patients and one caregiver) will provide quarterly consultative input on participant burden and lay dissemination; see Methods, Patient and public involvement, for details.

### Composition of the data monitoring committee, its role and reporting structure {21a}

The DSMB will review unblinded safety data every 6 months (and ad hoc if needed) and provide recommendations to the TSC and Sponsor/PI; no formal interim efficacy analysis is planned.

### Adverse event reporting and harms {22}

All adverse events reported by the subject or observed by the investigator will be recorded. There were no serious adverse reactions to rTMS treatment. Some of the milder adverse events included headache, fatigue, and mild dizziness, and these adverse events usually resolved on their own at the end of treatment. Causality of study treatment events will be documented. Some complications are considered Adverse Events of Special Interest (AESI): e.g., fever, bleeding, wound infection, pneumonia, and deep vein thrombosis. Serious adverse events should be promptly reported to the Ethics Committee of Shanghai Fourth People’s Hospital. All AEs/SAEs will be captured and coded; rTMS-related events will be specifically adjudicated for relatedness and seriousness. Any event meeting the DSMB safety triggers will prompt an ad hoc DSMB review, with possible recommendations to pause, modify, or terminate the trial.

### Frequency and plans for auditing trial conduct {23}

Following the detailed audit guidelines for research projects at Shanghai Fourth People’s Hospital, a Research Supervisor has been appointed to oversee the management of these projects. This supervisor will be responsible for conducting at least two site visits per year to verify the existence and completeness of all investigative documents in the research program. In addition, in order to further improve the quality of the study, the supervisor will randomly select a 25% sample of patients and conduct a detailed data review to ensure that each patient’s informed consent form has been properly signed and filed, to rigorously validate that the inclusion and exclusion criteria for the patients meet the established requirements, to verify the accuracy and traceability of the source data, and to focus on the absence of Adverse Events (AEs) and the effectiveness of the timely reporting mechanism. reporting mechanisms. For a more detailed review of the process and requirements, please refer to the hospital’s Research Monitoring Program document.

### Plans for communicating important protocol amendments to relevant parties (e.g., trial participants, ethical committees) {25}

Any substantial modification of this study will be immediately and fully notified to the Ethics Review Committee and the management of Shanghai Fourth People’s Hospital to ensure transparency and compliance. Non-substantive adjustments will be recorded and filed in detail for easy traceability. If the modification affects the participants, they will be informed in a timely manner. If necessary, additional consent will be obtained and registered in accordance with the law. At the same time, online trial registration information will be synchronized and updated.

### Dissemination plans {31a}

All results of this study, including positive positive results and negative results that failed to meet the pre-determined objectives, will be submitted in a full and fair manner to internationally recognized peer-reviewed journals to ensure full and transparent disclosure of the research results.

## Discussion

The incidence of POCD is higher among patients undergoing TKA, with clinically meaningful consequences for recovery and quality of life. Zhou et al. reported that perioperative systemic and neuroinflammation can injure neuronal populations, particularly in the hippocampus, and are accompanied by elevated cytokines including TNF-α, IL-1, IL-3, IL-4, IL-5, IL-6, IL-10, IL-12, and IL-17, which are implicated in the pathophysiology of POCD [19]. Although the precise mechanisms remain incompletely understood, these findings reinforce neuroinflammation as a plausible therapeutic target in the perioperative setting.

Beyond conventional medications, routine rehabilitation, and cognitive training, which can improve cognition to a degree, adding non-invasive brain stimulation with rTMS has been associated with greater cognitive gains in other contexts [20]. From a mechanistic standpoint, DLPFC-targeted rTMS may modulate perioperative neuroimmune signaling by reducing the release of pro-inflammatory cytokines such as IL-6 and TNF-α, attenuating microglial activation, and thereby limiting downstream synaptic dysfunction. rTMS has also been linked to enhancement of synaptic plasticity and strengthening of prefrontal–hippocampal network dynamics [21]. Together, these effects provide a biologically plausible route to support early postoperative cognition. Consistent with this rationale, we selected a pre-conditioning schedule of left DLPFC stimulation before surgery and paired clinical cognitive outcomes with inflammatory biomarkers, allowing us to explore whether cognitive changes track with cytokine dynamics in the immediate postoperative period.

Compared with prior work, the present trial advances the field in three ways. First, it focuses on a high-risk surgical population of older adults undergoing TKA, in whom POCD is common and clinically consequential. Second, it employs a sham-controlled, assessor-blinded design with a brief preoperative rTMS course to minimize early postoperative confounding. Third, it integrates clinical scales with a blood biomarker panel to enable mechanistic inference on neuroinflammatory pathways alongside cognitive endpoints. These design elements aim to address limitations of earlier studies that either lacked neuromodulatory interventions or did not concurrently assess inflammation and cognition.

The study also has limitations. It is single-center with a modest sample size and a short follow-up window, which may limit external validity and preclude conclusions about durability. Although perioperative care is standardized, residual confounding from unavoidable variations in analgesic and antiemetic regimens cannot be fully excluded despite prospective recording and covariate adjustment. In addition, there is no field-wide consensus on optimal rTMS parameters for postoperative cognition, including coil target, trains, pulses per session, and session frequency; our protocol tests one biologically informed option but not the full parameter space.

Future work should be concrete and hypothesis-driven. If the present results are positive, a multi-center confirmatory trial should (1) optimise dose and schedule (train number, intensity, timing relative to surgery), (2) extend follow-up to 1–3 months to assess durability and patient-centered outcomes, and (3) incorporate mediation analyses to test whether cytokine changes statistically explain cognitive benefit. Subgroup analyses by age or baseline cognitive reserve may clarify heterogeneity of treatment effect, and optional mechanistic sub-studies using EEG or functional neuroimaging could validate network engagement. If results are null, parameter refinement (targeting, intensity, timing) and outcome sensitivity should be interrogated before deprioritizing rTMS for POCD prevention.

In conclusion, if rTMS meaningfully improves early postoperative cognition in older adults after TKA, it would support the development of non-pharmacological, neuromodulation-based adjuncts within enhanced recovery pathways to mitigate or even arrest postoperative cognitive decline.

### Trial status

The study was registered in the Chinese Clinical Trial Registry (ChiCTR2400081372) on 29 February 2024 and is currently in the patient enrollment and data collection phase. Patient enrollment commenced on 10 March 2024 and is expected to be completed by 29 February 2026. If there are any changes in the protocol, we will notify the investigators, subjects, and other relevant persons by email or phone. This protocol is 1st Version and dated 1 April 2024.

## Supplementary Information

Supplementary Material 1.

## Data Availability

The datasets used and/or analyzed in this study are available to the corresponding author, Xiaohua Ke, upon reasonable request.

## Abbreviations

TKA: Total knee arthroplasty
POCD: Postoperative cognitive dysfunction
DLPFC: Dorsolateral prefrontal cortex
rTMS: Repetitive transcranial magnetic stimulation
MoCA: Montreal Cognitive Assessment
DST: Digit Span Test
DSST: Digit Symbol Substitution Test
TMT: Trail Making Test
DSR: Delayed Story Recall
IL-1β: Interleukin-1β
IL-6: Interleukin-6
TNF-α: Tumor necrosis factor-α
HMGB1: High-mobility group box 1
VTE: Venous thromboembolism
ERAS: Enhanced recovery after surgery
ITT: Intention-to-treat
eCRF: Electronic case report form
RMT: Resting motor threshold
MEP: Motor-evoked potential
MME: Morphine milligram equivalents
TCC: Trial Coordination Centre
TSC: Trial Steering Committee
AE: Adverse event
SAE: Serious adverse event
DSMB: Data and Safety Monitoring Board

## References

[1] Zeng L, Zhou G, Yang W, Liu J. Guidelines for the diagnosis and treatment of knee osteoarthritis with integrative medicine based on traditional Chinese medicine. Front Med (Lausanne). 2023;10:1260943. 37915321 10.3389/fmed.2023.1260943 PMC10617515

[2] Chen AT, Bronsther CI, Stanley EE, Paltiel AD, Sullivan JK, Collins JE, et al. The value of total knee replacement in patients with knee osteoarthritis and a body mass index of 40 kg/m2 or greater : a cost-effectiveness analysis. Ann Intern Med. 2021;174(6):747–57. 33750190 10.7326/M20-4722 PMC8288249

[3] Omara AF, Mohsen HH, Mohammed Abo Hagar A, Abdelrahman AF. Intrathecal Morphine versus Morphine-Dexmedetomidine Combination for Postoperative Pain Control After Total Knee Replacement: A Randomized Controlled Trial. Local Reg Anesth. 2023;16:113-122. 10.2147/LRA.S419465 PMC10404426 37551367

[4] Anastasio AT, Kim BI, Cochrane NH, Belay E, Bolognesi MP, Talaski GM, et al. Higher risk of reoperation after total knee arthroplasty in young and elderly patients. Materials. 2023;16(21):7012. 37959609 10.3390/ma16217012 PMC10648704

[5] Wang X, Zhou J, Zhang G. Effects of conventional nursing in the operating room combined with transcutaneous electrical acupoint stimulation on postoperative cognitive dysfunction after total knee arthroplasty in elderly patients. J Orthop Surg Res. 2024;18(1):906. 38297396 10.1186/s13018-023-04348-6 PMC10832165

[6] Zhu Q, Huang Y, Zhu X, Peng L, Wang H, Gao S, et al. Mannose-coated superparamagnetic iron oxide nanozyme for preventing postoperative cognitive dysfunction. Mater Today Bio. 2023;19:100568. 36846307 10.1016/j.mtbio.2023.100568 PMC9945786

[7] Kitsis P, Zisimou T, Gkiatas I, Kostas-Agnantis I, Gelalis I, Korompilias A, et al. Postoperative delirium and postoperative cognitive dysfunction in patients with elective hip or knee arthroplasty: a narrative review of the literature. Life (Basel). 2022;12(2):314. 35207601 10.3390/life12020314 PMC8878498

[8] Hu L, Luo M, Huang H, Wu L, Ouyang W, Tong J, et al. Perioperative probiotics attenuates postoperative cognitive dysfunction in elderly patients undergoing hip or knee arthroplasty: A randomized, double-blind, and placebo-controlled trial. Front Aging Neurosci. 2022;14:1037904. 36688164 10.3389/fnagi.2022.1037904 PMC9849892

[9] Zhang M, Yin Y. Dual roles of anesthetics in postoperative cognitive dysfunction: regulation of microglial activation through inflammatory signaling pathways. Front Immunol. 2023;14:1102312. 36776829 10.3389/fimmu.2023.1102312 PMC9911670

[10] Peng W, Lu W, Jiang X, Xiong C, Chai H, Cai L, et al. Current progress on neuroinflammation-mediated postoperative cognitive dysfunction: an update. Curr Mol Med. 2023;23(10):1077–86. 36411553 10.2174/1566524023666221118140523

[11] Saxena S, Kruys V, De Jongh R, Vamecq J, Maze M. High-mobility group box-1 and its potential role in perioperative neurocognitive disorders. Cells. 2021;10(10):2582. 34685561 10.3390/cells10102582 PMC8533835

[12] Terrando N, Yang T, Wang X, Fang J, Cao M, Andersson U, et al. Systemic HMGB1 neutralization prevents postoperative neurocognitive dysfunction in aged rats. Front Immunol. 2016;7:441. 27822212 10.3389/fimmu.2016.00441 PMC5075578

[13] Wei Z, Fu J, Liang H, Liu M, Ye X, Zhong P. The therapeutic efficacy of transcranial magnetic stimulation in managing Alzheimer’s disease: a systemic review and meta-analysis. Front Aging Neurosci. 2022;14:980998. 36147701 10.3389/fnagi.2022.980998 PMC9485622

[14] Tan S, Chen W, Kong G, Wei L, Xie Y. Peripheral inflammation and neurocognitive impairment: correlations, underlying mechanisms, and therapeutic implications. Front Aging Neurosci. 2023;15:1305790. 38094503 10.3389/fnagi.2023.1305790 PMC10716308

[15] Safavynia SA, Goldstein PA. The role of neuroinflammation in postoperative cognitive dysfunction: moving from hypothesis to treatment. Front Psychiatry. 2018;9:752. 30705643 10.3389/fpsyt.2018.00752 PMC6345198

[16] Luo A, Yan J, Tang X, Zhao Y, Zhou B, Li S. Postoperative cognitive dysfunction in the aged: the collision of neuroinflammaging with perioperative neuroinflammation. Inflammopharmacology. 2019;27(1):27–37. 30607668 10.1007/s10787-018-00559-0

[17] Newman SP. Analysis and interpretation of neuropsychologic tests in cardiac surgery. Ann Thorac Surg. 1995;59(5):1351–5. 7733767 10.1016/0003-4975(95)00215-7

[18] Rodriguez RA, Tellier A, Grabowski J, Fazekas A, Turek M, Miller D, et al. Cognitive dysfunction after total knee arthroplasty: effects of intraoperative cerebral embolization and postoperative complications. J Arthroplasty. 2005;20(6):763–71. 16139714 10.1016/j.arth.2005.05.004

[19] Zhou Y, Ju H, Hu Y, Li T, Chen Z, Si Y, et al. Tregs dysfunction aggravates postoperative cognitive impairment in aged mice. J Neuroinflammation. 2023;20(1):75. 36932450 10.1186/s12974-023-02760-7 PMC10022212

[20] Li H, Ma J, Zhang J, Shi WY, Mei HN, Xing Y. Repetitive transcranial magnetic stimulation (rTMS) modulates thyroid hormones level and cognition in the recovery stage of stroke patients with cognitive dysfunction. Med Sci Monit. 2021;27:e931914. 34686649 10.12659/MSM.931914 PMC8549488

[21] Zuo C, Cao H, Feng F, Li G, Huang Y, Zhu L, et al. Repetitive transcranial magnetic stimulation exerts anti-inflammatory effects via modulating glial activation in mice with chronic unpredictable mild stress-induced depression. Int Immunopharmacol. 2022;109:108788. 35504201 10.1016/j.intimp.2022.108788

