# Supplementary material for: Effect of perioperative repetitive transcranial magnetic stimulation on postoperative cognitive function and peripheral inflammation in elderly total knee arthroplasty patients: study protocol for a randomized controlled trial
Source: Trials. 2025 Oct 15;26:414. doi: 10.1186/s13063-025-09158-1 (PMC12523019; doi:10.1186/s13063-025-09158-1)
Supplement: Supplementary file 1 — Supplementary Material 1. [file 13063_2025_9158_MOESM1_ESM.docx]

**Informed Consent Form - Information Page**

**Version Number:** 1.0

**Date:** April 1, 2024

**Ethical Approval Number:** SYLL2023148

**Dear Sir/Madam:**

We would like to invite you to participate in a clinical trial study titled "Effect of perioperative repetitive transcranial magnetic stimulation on postoperative cognitive function and peripheral inflammation in elderly total knee arthroplasty patients: a randomised controlled trial".

Before you decide whether to participate in this study, please read the following content carefully. It can help you understand the content of the study, why this study is conducted, and the potential benefits, risks and discomforts that this study may bring to you. This study has been reviewed by the Ethics Review Committee of Shanghai Fourth People's Hospital Affiliated to Tongji University. It complies with relevant Chinese regulations and the ethical principles of protecting the rights and interests of subjects as stipulated in the Helsinki Declaration.

**Background**

Total-knee-replacement (TKA) is an effective treatment for end-stage osteoarthritis, which can alleviate symptoms such as knee joint pain, deformity, and movement disorders. Although TKA is the most effective treatment method, it is also considered one of the most invasive and painful orthopedic surgeries, making elderly patients prone to various postoperative complications. Among them, postoperative cognitive dysfunction (POCD) is a common central nervous system complication after surgery, mainly referring to functional impairments in language memory, visual memory, attention, language comprehension ability, and social skills that occur after anesthesia and surgery. Currently, there are no effective intervention measures for this condition. Repetitive transcranial magnetic stimulation (rTMS) is a painless and non-invasive cortical stimulation method with advantages such as simple operation and safety. It has been widely used in clinical practice in recent years. TMS generates complex neuro-biochemical effects, such as regulation of the neuroendocrine system, reduction of oxidative stress and neuroprotective effects of inflammation, and increase in the expression of neurotrophic factors. These molecular effects can reprogram the expression of inhibitory and excitatory neurotransmitters and their receptors of the same origin, and may also change the extrinsic and intrinsic electrophysiological characteristics of neurons, thereby leading to similar persistent synaptic plasticity changes as long-term potentiation (LTP) and long-term depression (LTD), ultimately helping to improve cortical neural regeneration or promoting neural repair, and possibly improving cognitive function.

**Objectives**

To clarify the clinical efficacy of perioperative TMS treatment in improving POCD in TKA patients, and further elucidate the inflammatory mechanism by which TMS alleviates POCD in TKA patients, thereby providing a theoretical basis for the clinical promotion and application of rTMS in the perioperative period.

**If I participate in the study, what will I need to do?**

1. Before you are selected for the study, the doctor will inquire about and record your medical history, and conduct a physical function examination. If you meet the inclusion criteria, you can voluntarily participate in the study and sign this informed consent form. If you do not wish to participate in the study, we will provide treatment according to your wishes, without affecting your scheduled TKA and routine perioperative care.

2. If you voluntarily participate in the study, the following steps will be followed: This study is a randomized, three-arm (conventional treatment group/TMS group/fake rTMS group), with a 1:1:1 allocation. You will be randomly assigned to one of these groups by computer. You and the doctor conducting the result evaluation will not know which group you have been assigned to (blinded); the rTMS operator knows the group assignment but will not participate in the result evaluation and data analysis. + The study period is from the start of treatment to 7 days after the treatment. During the study, the doctor will record your medical history and functional assessment results. The content and specific steps of each examination will be explained by your therapist and physician.

3. Evaluation indicators

Some of the tests in this study, such as cognitive function assessment and inflammatory index detection, require your cooperation to complete. Therefore, you may feel nervous. To accurately complete these tests, please follow the doctor's instructions.

**What are the inclusion criteria and exclusion criteria?**

1. Inclusion criteria
2. age ≥ 60 years, male or female;
3. needed to be performed TKA due to knee osteoarthritis;
4. American Society of Anesthesiologists grade I to II;
5. Preoperative Montreal Cognitive Assessment (MoCA) ≥ 26 points;
6. normal speech and hearing, able to co-operate with rehabilitation assessment and rehabilitation therapy.
7. Exclusion Criteria
   1. drug allergy, severe cardiovascular disease, hepatic or renal dysfunction;
   2. use of sedative-hypnotics or medications with anticholinergic effects that cannot be discontinued at least 4 weeks prior to baseline;
   3. contraindications to rTMS (e.g., history of epilepsy, cochlear implant recipients, patients with metal implants in the head or pacemakers in the heart);
   4. those who are participating in other clinical trials;
   5. postoperative complications such as lung 34 infection, pulmonary embolism, or stroke;
   6. personal or legal representatives unable or unwilling to give written informed consent.

**What benefits can you expect from participating in this study?**
Participating in this study may improve your walking ability, or it may not.
**What risks are associated with my participation in the study?**
Common discomforts caused by rTMS include: local discomfort/stinging sensation on the scalp, brief headache or dizziness, tinnitus/noise discomfort (usually mild to moderate, and temporary and can be relieved). We will provide you with earplugs/ear covers and provide continuous monitoring throughout the process. An rTMS/sham session will be interrupted or discontinued if any of the following occur during or immediately after stimulation: (1) intolerable scalp discomfort or pain despite repositioning; (2) vasovagal syncope or pre-syncope; (3) sustained hypertension (>180/110 mmHg), tachycardia (>130 bpm) or clinically significant arrhythmia; (4) new focal neurological symptoms/signs; (5) any seizure; (6) coil-related skin heating/burn; or (7) persistent severe headache not relieved by simple analgesia, or any other event judged clinically significant and related to the procedure by the investigator. The session will be stopped, clinical care provided, and the event documented and reported per the Harms procedures. Resumption of stimulation at a reduced intensity and/or fewer trains may be considered after resolution of symptoms at the investigator’s discretion. Occurrence of a seizure or any unanticipated serious adverse device effect will lead to permanent discontinuation of rTMS for that participant. Participants may also withdraw from the intervention at any time; unless consent is withdrawn, they will remain in outcome follow-up (intention-to-treat). Trial-level pausing or termination: Decisions to pause, modify, or terminate the trial will be guided by the independent DSMB according to pre-specified safety triggers and overall safety review Interim analyses and stopping guidelines and Harms). Illustrative triggers include: any definite device-related seizure; ≥2 rTMS-related SAEs or an rTMS-related SAE rate >5% and exceeding the sham arm by ≥5 percentage points; any unanticipated serious adverse device effect; or a clear excess of all-cause SAEs in the active rTMS arm at a scheduled review. The DSMB will issue recommendations to the TSC and Sponsor/PI; final operational decisions will be made by the Sponsor/PI upon TSC advice.

If you have a history of epilepsy, metal implants, cardiac pacemakers/defibrillators, cochlear implants, etc., please inform your doctor in advance. If you experience any discomfort, or if your condition changes in any way, or if there is any unexpected situation during the study, regardless of whether it is related to the study, you should promptly notify your therapist. He/She will make a judgment and provide appropriate medical treatment. During the study, your other treatments are not restricted. You have the right to decide to withdraw from this study at any time, whether you decide to participate or refuse to participate in this study, it will not affect the relationship between you and your therapist. In addition, there may be a risk of privacy leakage in this clinical study. We will take a series of measures to protect your privacy, such as: only your initials will appear in the case report form, and no identifiable information will be disclosed.

**Will participating in this research increase my medical expenses?**

The cognitive function assessment and inflammation index tests you undergo after being enrolled are free. The rTMS treatment you receive after being in the experimental group is also free.
**What compensations will I receive for participating in this research?**

Due to the limited funds of the project, you will not receive any economic compensation for participating in this research.
**Damages and Compensation**

If you suffer any injuries related to this research, our research group will compensate and provide remedies in accordance with the relevant laws and regulations of the country. For consultation on handling and compensation related to research-related injuries, please contact: Xiaohua Ke, Tel: 16621719878, Email: kxh22@tongji.edu.cn.

**Is personal information kept confidential?**

All the information you provide for this study will be recorded in the research case file/case report form. All the test results appearing in the original medical records (including personal information, test reports, etc.) will be fully confidential within the legal limits. Your name will not be included in the CRF form; only your name in the pinyin abbreviation and the number assigned to you during the trial participation will be shown. In relevant research summaries, articles, and public publications, if necessary, only your pinyin abbreviation and the number will be presented.

When required, the ethics committee or the project funding department can access the participant's information for the study. However, without permission, they will not use or disclose the participant's information for other purposes or to other organizations.
Data will be stored for at least 5 years; when published or made public, it will only be presented in an aggregated form and cannot identify individuals.
**How to obtain more information?**

You can raise any questions about this trial study at any time and consult Dr. Xiaohua Ke. The contact number is 16621719878 and the email is kxh22@tongji.edu.cn. If there is any important new information during the trial that may affect your willingness to continue participating in the study, your doctor will notify you in time.
**Must I participate in this study or can I withdraw at any time?**

Whether to participate in this study is entirely up to your voluntary choice. You can refuse to participate in this study. At any time during the study, you have the right to withdraw from the study without giving a reason. If you refuse to participate or withdraw at any time, your benefits will not be affected, and you will not be discriminated against or retaliated against. If you choose to participate in this study, we hope you can complete all the trial procedures.
If you withdraw your consent, the de-identified data collected will continue to be used for scientific analysis to ensure the integrity and reliability of the research results (if you do not agree, you can communicate with the study doctor).

Your doctor or researcher, considering your best interests, may terminate your participation in this study at any time.

**Biological Samples (Blood Collection) and Their Uses**

This study will collect approximately 5 mL of blood at the baseline/3rd day after surgery/7th day for the detection of inflammatory indicators (IL-1β, IL-6, TNF-α, HMGB1). The samples will only be used for this study. They will be stored at -80 °C for a maximum of 10 years and will be destroyed upon expiration or at the end of the study. They will not be used for other studies (if you are willing to authorize future related studies, please check the separate consent item). Agree □

**What should be done now?**

Whether to participate in this trial study is up to you. You can discuss it with your family or friends before making a decision.

Before making the decision to participate in the trial, please try to ask your doctor as many questions as possible until you fully understand this trial study.

**Ethics Committee**

If you have any dissatisfaction in the study, please contact the Ethics Review Committee of Shanghai Fourth People's Hospital Affiliated to Tongji University.

Ethics Committee Office: Ethics Committee Office on the 7th floor of the Outpatient Building of Shanghai Fourth People's Hospital Affiliated to Tongji University.

Contact Number: 021-55603023.

Thank you for reading the above materials. If you decide to participate in this trial study, please tell your doctor, and he/she will arrange all matters related to the trial for you.

Please keep this document.

**Informed Consent Form - Signature Page**

**Consent Statement**

1. I have read this informed consent form, and the relevant personnel of the project have explained the purpose, content, risks and benefits of this trial to me in detail.

2. I have discussed and asked about the relevant questions of this research, and the answers to these questions have satisfied me.

3. I have sufficient time to make a decision.

4. I voluntarily agree to participate in the clinical research described in this statement.

5. If I need to withdraw from this product due to its reasons, I will promptly inform the doctor of the changes in my condition.

6. If due to changes in my condition, I need to take any other treatment, I will seek the doctor's opinion in advance or inform the doctor truthfully afterwards.

7. I agree that the drug supervision and administration department, the ethics committee or the project funding department representatives can review my research materials.

8. I will receive a copy of the informed consent form signed and dated.

9. After careful consideration, I voluntarily participate in "The Effect and Mechanism of Perioperative Repetitive Transcranial Magnetic Stimulation on the Early Cognitive Function of Elderly Patients Undergoing Total Knee Arthroplasty". If I have any questions about the research and the rights and interests of the subjects, or if there are any trial-related damages, I can contact the following researchers: Xiaohua Ke, phone: 16621719878, email: kxh22@tongji.edu.cn.

Finally, I decide to agree to participate in this trial study and guarantee to follow the doctor's advice.

Subject's Signature: Date: Year Month Day

Subject's Contact Number:

Legal Representative's Signature: Date: Year Month Day

Relationship with the Subject: Legal Representative's Phone: _______________________________________

I confirm that I have explained the detailed situation of this research to the subject, including their rights and possible benefits and risks, and have given them a signed copy of the informed consent form.

Doctor's Signature: Date: Year Month Day

Doctor's Contact Information:

(This page is a necessary part of the subject's informed consent form. Each "subject's informed consent form" must have the signatures and dates of the subject or legal representative and the doctor to be valid.)
